# Volumes and velocities: Meta-analysis of PC-MRI studies in normal pressure hydrocephalus

**DOI:** 10.1007/s00701-024-06333-2

**Published:** 2024-11-19

**Authors:** Helen Whitley, Petr Skalický, Awista Zazay, Adéla Bubeníková, Ondrej Bradáč

**Affiliations:** 1 Department of Neurosurgery and Neurooncology, Military University Hospital and Charles University, U Vojenské Nemocnice 1200/1, 162 00 Prague 6, Czech Republic; 2 Department of Neurosurgery, 2nd Faculty of Medicine, Charles University in Prague and Motol University Hospital, Prague, Czech Republic; 3 Charing Cross Hospital, Imperial College Healthcare, London, UK; 4 First Faculty of Medicine, Charles University, Nové Město, Czech Republic

**Keywords:** Normal pressure hydrocephalus, Phase contrast magnetic resonance imaging, Neuroimaging, Cerebrospinal fluid, Stroke volume, Peak velocity

## Abstract

**Purpose:**

Phase contrast magnetic resonance imaging (PC-MRI) represents an opportunity to non-invasively investigate cerebral spinal fluid (CSF) flow in patients with idiopathic normal pressure hydrocephalus (iNPH). Studies in recent years have explored the diagnostic and prognostic value of PC-MRI derived parameters. This review aims to identify all PC-MRI studies of iNPH published since 2010, synthesise a review based on collated results, and analyse specific flow parameters identified in the selected studies.

**Methods:**

Our protocol was prospectively registered on PROSPERO [CRD42020180826]. We systematically searched four databases: Pubmed, Web of Science, Ovid, and Cochrane library to identify all eligible studies. Quality assessment was performed using a modified Newcastle–Ottawa Scale [19]. Systematic review was conducted according to Prisma guidelines. A random-effects model was used to perform meta-analysis on the available flow parameters.

**Results:**

Eighteen records were identified for inclusion. Five studies were eligible for meta-analysis, representing 107 iNPH patients and 82 controls. CSF flow parameters available for analysis were stroke volume and peak velocity. Both were significantly higher than controls (p = 0.0007 and p = 0.0045 respectively) according to our random-effects analysis, consistent with a model of hyper-dynamic CSF in iNPH. Our systematic review revealed average stroke volumes in iNPH ranging from 43uL to over 200uL. Peak velocity values ranged from 5.9 cm/s to 12.8 cm/s.

**Conclusion:**

Significant increases in stroke volume and peak velocity values in iNPH patients suggest a place for PC-MRI as supplementary evidence in the diagnostic work-up of iNPH. Although shunting reduces aqueductal stroke volume and peak velocity, the ability of pre-shunt values to reliably predict treatment response remains complicated. We suggest that it may be more appropriate to consider a range of values that reflect varying probabilities of shunt success. We recommend that future studies should prioritise standardising PC-MRI protocols, and before then PC-MRI findings should be considered supportive rather than determinative.

**Supplementary Information:**

The online version contains supplementary material available at 10.1007/s00701-024-06333-2.

## Introduction

Phase-contrast Magnetic Resonance (PC-MRI) imaging is a type of velocimetry sensitive to flow. Phase data is intrinsic to all MRI signals, and in PC-MRI this signal is used to derive velocity (cm/s) and flow (μL/cardiac cycle). This sensitivity makes PC-MRI a useful tool for studying cerebral spinal fluid (CSF) dynamics. Highlighting abnormal CSF flow patterns may aid in diagnosis of disorders such as idiopathic normal pressure hydrocephalus (iNPH).

While Moran et al.first suggested the clinical use of PC-MRI in 1985 [28], its application to CSF dynamics began in 1987 with Feinberg and Mark’s et al.’s study on cardiac-gated MRI, providing evidence that vascular-driven brain movement during systole drives CSF flow [14]. This was built upon by Enzman in 1991 who measured CSF velocity at various anatomical sites across the brain and spinal canal, finding that flow starts with carotid systole, varies between subarachnoid and ventricular spaces, and suggested the fourth ventricle and distal spine as spaces for mixing and capacitance [13]. In 1993, a PC-MRI study by Thomas et al. explored CSF dynamics in hydrocephalus, suggesting that flow dynamics could be correlated with ICP to assess brain compliance [29]. The first to suggest that PC-MRI could be used in iNPH diagnostics was Bradley et al. in their frequently cited 1996 paper describing a relationship between shunt responsiveness stroke volumes above 42uL [10]. The concept of CSF oscillations as a function of arterial and venous blood flow was brought to the field by Greitz, who used PC-MRI data to demonstrate increased systolic pulse pressure in chronic hydrocephalus [15, 16]. Important concepts were also brought to the field by Bateman, who proposed that patients with NPH have reduced venous compliance [8], and Balédent, who in 2001 revealed that CSF flow timing is sequentially co-ordinated across ventricular regions [5]. In 2004 the same group built upon this by showing that in hydrocephalus patients, peak jugular flow occurs earlier in the cardiac cycle, and hyperdynamic CSF flow is seen in the cerebral aqueduct [6]. Noam Alperin introduced the CSF pressure gradient waveform in 2004, using PC-MRI to study pulsatile flow [2].Together, these insights suggest that CSF flow measurements can help detect pathological flow changes.

Against this background, PC-MRI may have the potential to reveal diagnostic biomarkers or predict shunt response in iNPH patients. Over at least the last decade, many studies have tried to show this. Our review assesses the evidence for PC-MRI to aid diagnosis and predict shunt response in these patients. iNPH is a relatively common disease, with a prevalence of between 3—6% [3, 17, 21]. and incidence that increases exponentially with age [20, 23]. In our review we collate and assess all studies published since 2010 that use PC-MRI derived CSF stroke volume and peak velocity in investigations of iNPH. We have compiled a narrative of collated results, and performed a meta-analysis of studies that used comparable methods to derive parameters. To our knowledge this is the first meta-analysis of PC-MRI data from imaging studies of patients with iNPH.

## Materials and Methods

Our protocol is available on the international PROSPERO database (CRD42020180826).

Our search strategy, data extraction and systematic review were conducted according to PRISMA guidelines.

### Search Strategy

To identify relevant material, we conducted a systematic search of all papers published from January 2010 to January 2023. Papers published earlier than January 2010 were excluded in order to reduce heterogeneity that may be introduced due to changes in signal processing of PC-MRI data. We searched the following academic databases: PubMed, Ovid, Web of Science, and Cochrane Library. Two authors conducted the search independently (HW and PS). Keywords searched were “Phase Contrast MRI” and “Normal Pressure Hydrocephalus”. Related terms (for example “Magnetic Resonance Imaging) were included in the search according to search engine algorithms.

Our criteria for inclusion were as follows: adults with a diagnosis or probable diagnosis of idiopathic normal pressure hydrocephalus (iNPH), investigated with PC-MRI. The studies had to measure CSF stroke volume and peak velocity in the cerebral aqueduct, and these CSF parameters had to be obtained using standardised methods. Stroke volume is defined as the total volume of CSF displaced during a single cardiac cycle, representing the sum of the forward (systolic) and reverse (diastolic) flow components in absolute terms. This provides the volume displaced during one cardiac cycle. This method is widely used due to its straightforward calculation and reliable results, making it a reliable consistent measure of CSF stroke volume.

Net flow was not considered in this study due to the high potential for error and the wider range of variation in how the value is derived. In principle, net flow is calculated by subtracting the caudocranial from the craniocaudal flow, and can lead to inaccuracies as it involves the challenge of subtracting large opposing flow volumes during the systolic and diastolic phases [7, 10, 25]. To maintain consistency and comparability, we excluded measures of net or average flow. This point is expanded in our discussion.

Criteria for exclusion were: Children or adults with other forms of hydrocephalus (acquired, obstructive, and non-iNPH communicating forms, eg. ependymoma). Review articles and case reports were excluded. Studies were selected by two authors (HW and PS), and disagreements were discussed and resolved by a third independent reviewer (AZ). Studies that met our criteria underwent full text review, data extraction, and quality assessment. Data-extraction and quality assessment was performed in duplicate (HW and PS).

### Quality assessment

To rate the strength of our evidence, we used a modified Newcastle–Ottawa Scale (NOS), which scores each study across three categories: Selection, comparability, and outcome. The total possible score was 10 and studies scoring less than 5 were considered to have a high risk of bias [43]. Our modified NOS scoring system can be found in Supplementary Table 1. Data on magnet strength, whether researchers were blinded, and the use of Relkin [33] guidelines were also extracted from each paper to inform the overall assessment. This data is included in Supplementary Table 2: Quality assessment and NOS scores. We used this data to assess heterogeneity of results and strength of evidence in each study.

### Analysis

Studies that underwent meta-analysis had further inclusion criteria. These were: 1) Healthy controls used as the comparator group, matched for age and gender 2) CSF parameters stroke volume and peak velocity derived from PC-MRI data using standardised and comparable methods. Data extracted for meta-analysis were means and standard deviations. Authors of papers not reporting means and standard deviations were contacted for aggregate data in the appropriate format. Heterogeneity was assessed according to the I^2^ statistic. Given the relatively high level of heterogeneity, we selected a random effects model to analyse the data. Analyses were carried out using R with the open-source package ‘metafor’ [41].

## Results

### Characteristics of included studies

**Fig. 1:**
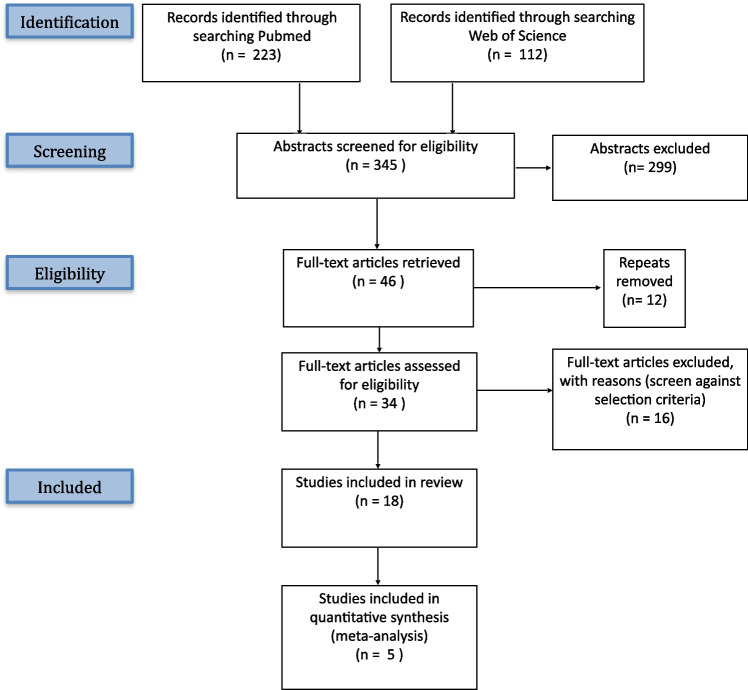
Prisma flowchart. Flow diagram illustrating the systematic study selection process for inclusion in the narrative review and meta-analysis, based on the Preferred Reporting Items for Systematic Reviews and Meta-Analyses (PRISMA) guidelines

Our initial search yielded a total of 345 abstracts, which were screened for eligibility against our inclusion and exclusion criteria. After excluding ineligible studies and removing repeats, 18 full texts were included in the review. These underwent full text analysis, quality assessment and data extraction. This process is summarised in Fig. 1: Prisma flowchart. The included studies comprised a total of 974 patients, including 473 with diagnosed or suspected iNPH. No new relevant articles were identified using citation tracking. Of the 18 included articles, 15 were cohort studies. Comparator groups varied according to the specific aims of each study. Comparators were either healthy control subjects, post-shunt iNPH patients, post CSF tap iNPH patients, or other non-NPH neurological patients. This data is presented in Table 1: Summary of included articles. A breakdown of the quality assessment is presented in Supplementary Table 2: Quality assessment and NOS Scores.

**Table 1:** Summary of included articles

Author (Date)	Study design	Cohort size	iNPH cases	Comparator group	NOS
Algin (2010)	Cohort	58	43	Healthy controls	9
Aslan, K. (2013)	Case–control	80	30	Healthy controls, post-shunt iNPH	9
Chen, C. (2022)	Cohort	216	38	Non-NPH neurological cases	10
Daouk, J. (2014)	Cohort	30	12	Alzheimers disease	7
Gokul, U, R. (2018)	Cohort	40	40	Post-CSF tapping NPH	7
Hamilton, R.B. (2019)	Cohort	42	30	Healthy controls, post-shunt iNPH	8
He, Wen-Jie (2020)	Cohort	107	40	Healthy controls, elderly aquired hydrocephalus	9
He, Wen-Jie (2022)	Cohort	46	46	Post-shunt iNPH	10
Lindstrom (2018)	Cohort	26	4	Healthy controls, post-shunt iNPH	9
Miskin, N. (2015)	Cohort	7	7	Post-shunt iNPH	7
Qvarlander, S (2017)	Cohort	51	16	Healthy controls	10
Shanks, J (2019)	Cohort	42	21	Post-shunt iNPH	10
Stecco, A. (2017)	Cohort	38	38	Post-shunt iNPH	6
Tawfik. (2017)	Observational	32	12	Healthy control, brain atrophy	7
Yamada, S (2020)	Cohort	73	41	Healthy controls and Alzheimers disease	7
Yin, L.K. (2017)	Cross-sectional	40	20	Healthy controls	9
Yousef, M.I. (2016)	Cohort	26	15	Healthy controls and brain atrophy	6
Witthiwej (2012)	Cohort	20	20	Post-shunt iNPH	5
		Total = 974	Total = 473		

Overview of the key characteristics and findings of the studies included in the review, detailing study design, sample size, and Newcastle Ottawa Scale score

### Meta-analysis

**Fig. 2:**
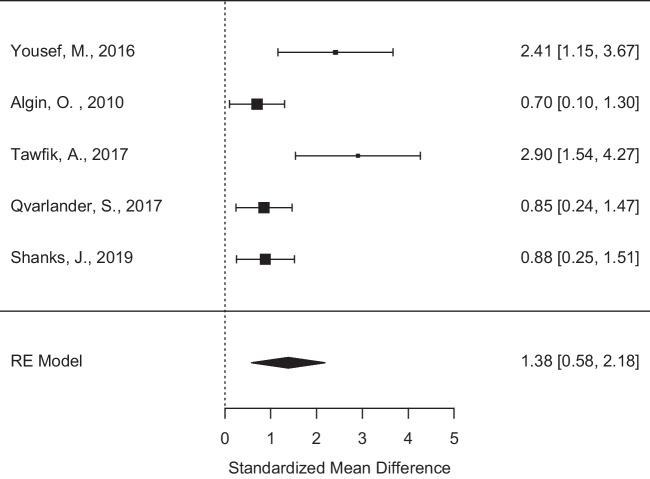
Random effects model of stroke velocity. Forest plot showing the standardised mean differences for stroke volume measurements between the iNPH and control groups across studies

**Fig. 3:**
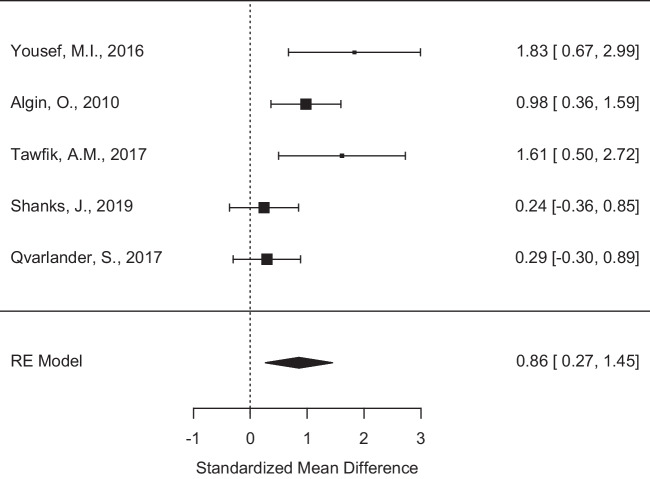
Random effects models of peak velocity. Forest plot showing the standardised mean differences for peak velocity measurements between the iNPH and control groups across studies

Five papers with data in the appropriate format were available to us for meta-analysis. This represented a total of 107 iNPH patients and 82 healthy controls. The parameters available for analysis were Stroke Volume and Peak Velocity. Heterogeneity tests were significant for both parameters. For stroke volume, I^2^ was 80.12% and p value = 0.0088. For peak velocity, I^2^ was 66.98% and p value = 0.0265. We used standardised mean difference (SMD) as a measure of effect size, and we selected a random-effects model on account of the high heterogeneity. Stroke volume was significantly higher than controls (p = 0.0007) in the random-effects model, with a SMD of 1.38, upper 95% CI 2.18 and lower 95% CI 0.58. (Fig. 2: Random effects model of Stroke volume.) Peak velocity was also higher in the iNPH group (p = 0.0045) with a SMD of 0.86, upper and lower 95% CI 0.27, 1.45 respectively. (Fig. 3: Random effects model of peak velocity). Units for the raw data were uL/cardiac cycle for stroke volume, and cm/s for peak velocity, transformed to SMD with the open-source package ‘metafor’ in R [41].

### CSF flow parameters

The following paragraphs summarise results of the flow parameters most frequently reported in our selective studies: stroke volume, and peak velocity. We provide a narrative review of findings, giving particular attention to potential clinical utility.

### Stroke volume

Stroke volume (uL) refers to the mean volume of CSF passing through a predefined space, typically the cerebral aqueduct, during one cardiac cycle. The parameter has gained attention as a diagnostic tool since Bradley et al. in 1996 identified a stroke volume of > 42uL as a marker of favourable outcomes post-shunting [10]. Subsequent studies have shown inconsistent results regarding its diagnostic utility.

Review of the literature shows that stroke volume results are generally higher in iNPH compared to age matched controls and compared to other neurological diseases [1, 11, 12, 18, 31, 35, 36]. Average values in iNPH ranged from 43.2uL [1] to 271uL [37], and in healthy subjects were as low as 3.9 uL [1]. Results are summarised in Table 2. Studies using net stroke volume, alternative units (e.g., µL/second rather than µL/cardiac cycle.), or ROIs other than the cerebral aqueduct are excluded as per our predefined criteria. Note that Tawfik et al. is not included in summary tables, as the study reports diagnostic accuracy, not average values.

**Table 2:** Summary of Stroke volume results

Author (Date)	Stroke Volume (uL)					
	iNPH		Comparator group			*p* value
	**mean ± SD**	**cohort size**		**mean ± SD**	**cohort size**	
Algin, O. (2010)	43.2 ± 63.8	*n* = 43	Healthy subjects	3.9 ± 3.9	n = 15	*p* < 0.5
Chen, C. (2022)	187.21 ± 144.31	*n* = 38	Non-iNPH neurological cases	103.19 ± 74.63	n = 178	*p* = 0.001 **
Daouk, J. (2014)	148.9 ± 72.2	*n* = 12	Alzheimers disease	45.4 ± 21.8	n = 18	*p* = 0.001 **
Qvarlander, S. (2017)	148 ± 95	*n* = 16	Healthy subjects	90 ± 50	n = 35	*p* = 0.01 *
Stecco (2017)	271,85 ± 143,03	*n* = 26 (improvement)	1 month post-shunt iNPH	213 ± 125,14	n = 26	
			6 months post-shunt iNPH	162,15 ± 91.5	n = 26	
	79,83 ± 31,24	*n* = 12 (no improvement)	1 month post-shunt iNPH	72,83 ± 28,66	n = 12	
			6 months post-shunt iNPH	62,83 ± 31,12	n = 12	
Yousef (2016)	105.12 ± 31.89	n = 15	Healthy subjects	27.26 ± 3.06	n = 5	
			Brain atrophy	17.7 ± 4.16	n = 6	
	**median [IQR] (range)**	**cohort size**		**median [IQR] (range)**	**cohort size**	
Hamilton, R.B. (2019)	124.5 [94.5] (37.1–275.0)	*n* = 26	Healthy subjects	49.7 [32.3] (23.1 – 90.5)	*n* = 10	*p* = 0.01 *
	144.6 [127.6] (37.1–249.6)	*n* = 9	Post-shunt iNPH	76.8 [55.1] (39.4 – 177.6)	*n* = 9	*p* = 0.001 **
He, Wen-Jie (2020)	19.5 [43.5]	*n* = 40	Aquired hydrocephalus	27.0 [58.5]	*n* = 41	NS
			Healthy subjects	15.0 [10.8]	*n* = 26	NS
He, Wen-Jie (2022)	11.3 [69.5]	*n* = 28 (improvement)				
	20.0 [74.0]	n = 18 (no improvement)				NS
Shanks, J (2019)	103.5 (69.8–142.8)	*n* = 21	Healthy subjects	62.5 (58.3 – 73.8)	*n* = 21	NS
Shanks, J (2019)	94.8 (81–241)	*n* = 21	Post-shunt iNPH	88 (51.8 – 173.3)	*n* = 21	NS
Yamada (2020)	49.0 (10–160)	*n* = 41 (upper aqueduct)	iNPH with AD	49.0 (10–160)	*n* = 23	NS
			Healthy subjects	18.9 (10–40)	*n* = 9	*p* < 0.001 **
	37.6 (10–140)	*n* = 41 (lower aqueduct)	iNPH with AD	39.6 (0–180)	*n* = 23	NS
Yin, L.K. (2017)	73.000 (42.125)	*n* = 20	Healthy subjects	23.500 (12.125)	*n* = 20	*p* = 0.001 *
			Healthy subjects	17.8 (0 – 40)	*n* = 9	*p* = 0.023 *

Comparison of stroke volume data across studies including means, standard deviations (SDs), or median and interquartile range (IQR) where available. Statistical significance is denoted as follows: * = *p* < 0.05; ** = *p* < 0.01; NS = Not Significant

Chen, C. et al. recruited 178 non-iNPH cases as controls, representing the largest cohort in this review. This study demonstrated that stroke volume is increased in iNPH compared to other neurological diseases with high significance (p < 0.001), and suggested that stroke volume provides better diagnostic accuracy than other PC-MRI parameters [11]. Repeatability of various flow parameters as assessed by Tawfik et al., concluded that stroke volume was the most reliable parameter with an accuracy of 100% [38].

Five selected studies compared stroke volume values before and after shunt placement [1, 18, 19, 35, 37]. Of these, only Stecco et al. concluded that there may be a place for PC-MRI in selecting patients for surgery [37]. In a study by Algin et al., there was no difference in stroke volume between patients who showed clinical improvement after shunting and those who did not. However, with a cohort of 18, the study may have lacked statistical power to detect a real effect [1]. Shanks et al. also showed no predictive value of CSF parameters, despite finding significantly elevated values compared to controls in the pre-operative group [35]. Wen Je He analysed the receiver operating characteristic (ROC) to analyse predictive value of CSF flow parameters. Comparing patients that showed improvement vs. no improvement post shunt, they demonstrated that area under the curve (AUC) of all CSF parameters was all close to 0.5, indicating no discriminatory power. However, among these, stroke volume was the least ineffective predictor, with an AUC of 0.62 [19]. Similar findings by Hamilton et al. [18] demonstrated a significant decrease in stroke volume post-shunting (p = 0.001), but the authors could not conclude that stroke volume predicts which patients would clinically benefit from the procedure. Interestingly, some patients showed clinical improvement even though their CSF volume did not decrease. Failure of the shunt was ruled out in these cases. An unchanged CSF volume therefore does not suggest the shunt is malfunctioning, nor does it predict whether the patient will experience clinical improvement following the procedure. The observed trend of decreased stroke volume ratio and CSF flow post-shunt aligns with other studies, and non-significant results may reflect variability in patient responses and limitations in statistical power, rather than an absence of a true effect.

Stecco et al. [37] investigated the use of stroke volume in predicting outcomes by dividing NPH patients into improved and unimproved groups post surgery. They found that patients with higher preoperative SV values (mean 271.85 ± 143.03) were more likely to show improvement, and those with lower (mean 79.83 ± 31.24) were less likely to show symptom improvement after surgery. The authors suggest that PC-MRU can be used as a useful pre-operative assessment tool. Although they did not suggest a cut-off SV value, higher pre-operative stroke volumes were associated with a greater likelihood of a positive surgical outcome.

### Peak velocity

Peak velocity (cm/s^−1^) represents the highest absolute velocity of CSF through the cerebral aqueduct in one cardiac cycle. In our selected studies, average values of peak velocity in iNPH patients ranged from 3.86 cm/s^−1^ [49] to 12.8 cm/s^−1^[18]. Studies that presented average peak velocity as mean ± SD or median ± IQR (and range, if available), are presented in Table 3. Peak velocity values are higher in iNPH compared to healthy controls, higher than non-iNPH neurological diseases, and higher than post-shunt iNPH cases. All studies measured peak velocity in the cerebral aqueduct.

**Table 3:** Summary of peak velocity results

Author (Date)	Peak Velocity (cm/s-1)					
	iNPH		Comparator group			*p* value
	**mean ± SD**	**cohort size**		**mean ± SD**	**cohort size**	
Algin (2010)	8.53 ± 4.13	*n* = 43	Healthy controls	4.78 ± 2.48	*n* = 15	*p* < 0.05 *
Chen, C. (2022)	9.956 ± 4.748	*n* = 38	Non-iNPH neurological cases	8.026 ± 3.350	*n* = 178	*p* = 0.038 *
Miskin (2015)	6.9 ± 3.6	*n* = 7	Post-shunt iNPH	8.3 ± 3.8	*n* = 7	
Witthiwej (2012)			Post-shunt iNPH	8.79 ± 2.76	*n* = 10 (Improvement)	*p* < 0.05 *
			Post-shunt iNPH	4.956 ± 1.889	*n* = 10 (No improvement)	
Tawfik (2017)	6.8 ± 1.4	*n* = 12	Healthy controls	4.2 ± 1.8	*n* = 6	*p* < 0.05 *
			Alzheimers disease	3.6 ± 1.4	*n* = 14	*p* < 0.05 *
Yousef (2016)	3.89 ± 1.236	*n* = 15	Healthy controls	2.274 ± 0.946	*n* = 5	*p* < 0.035 *
			Alzheimers disease	1.842 ± 0.959	*n* = 6	NS
	**median [IQR] (range)**	**cohort size**		**median [IQR] (range)**	**cohort size**	
Shanks, J (2019)	10.3 [9.8]	n = 21	Healthy controls	10.3 [3.4]	*n* = 21	NS
Hamilton, R.B. (2019)	12.8 [8.3] (5.3—21.4)	*n* = 26	Healthy controls	7.9 [4.5] (5.3—15.0)	*n* = 10	
Yin, L.K. (2017)	7.445 [4.32]	*n* = 10	Healthy controls	5.81 [2.19]	*n* = 20	
Wen-Jei He (2020)	5.374 [7.158]	*n* = 40	Healthy controls	5.8105(3.760)	*n* = 26	NS
			Elderly aquired hydrocephalus	6.641(8.760)	*n* = 41	NS
Wen-Jei He (2022)	3.775 [7.354]	*n* = 28 (Improvement after shunt)				
	5.937 [10.025]	*n* = 18 (No improvement after shunt)				

Comparison of peak velocity data across studies, including means, standard deviations (SDs), or median and interquartile range (IQR) where available. Statistical significance is denoted as follows: * = p < 0.05; ** = p < 0.01; NS = Not Significant

Chen et al. investigated a large cohort [n = 216] with non-iNPH neurological cases as the comparator group. They demonstrated significantly higher peak velocity values in iNPH (p = 0.0338), and suggested that peak velocity could aid in iNPH diagnosis due to its high specificity (90.4%) at a cutoff value of 11.65 cm/s^−1^ [11]. Tawfik et al. also found higher peak velocity values in iNPH compared to healthy controls and brain atrophy cases, reporting 82.7% diagnostic accuracy for peak systolic velocity [38]. Yousef et al. measured both peak and mean velocity in the systolic phase, also finding higher values in iNPH compared to controls. [49]. Yin et al. reported peak velocity in the systolic and diastolic phases separately, finding a significant (p = 0.001) difference between iNPH and control groups in the diastolic phase [48].

Despite promising results regarding the diagnostic utility of peak velocity, when attempting to predict shut response, results are inconsistent. Wen-Jie He et al. did not find any PC-MRI parameters that could identify shunt responders [19]. Similarly, Shanks et al. found no correlation between peak velocity and shunting outcomes, but also found no differences between iNPH and healthy controls [35]. Exceptionally, Miskin et al. found that peak velocity values increased after shunting [27]. However their study’s findings are limited with a cohort of just seven. Additionally, in this study the use of two different field strengths (3 T and 1.5 T) limits validity, as stroke volume values can be scanner and parameter dependent [26, 44]. 3 T MRI offers higher SNR, higher achievable spatial and temporal resolutions and usually comes with stronger gradient systems. This makes 3 T particularly beneficial for measuring flow through small-diameter structures (e.g. aqueduct) and at low speeds (e.g. CSF). However, it is also more susceptible to various types of artefacts.

A retrospective study by Witthiwej et al. found a higher pre-shunt peak velocity in the group that improved after shunting. They suggested a peak velocity threshold of 7.0 cm/s^−1^ to identify the responsive group, reporting a sensitivity of 60%, specificity 83.3%, positive predictive value 81.5% and accuracy 60% [45]. However, given limitations identified in study design, these metrics should be seen as preliminary rather than definitive. Generalisability is compromised without a mention of specific inclusion and exclusion criteria, and it is unclear how "clinically suspected" iNPH was defined. They report on 20 patients, without discussion on the study’s statistical power. The threshold selection of 7.0 cm/s^−1^ may therefore be vulnerable to overfitting, resulting in misleadingly high specificity. A mean follow-up period of 6.6 months may not be long enough to capture long-term outcomes. These limitations contributed to an NOS score of 5/10, reflecting issues in selection criteria, comparability and followup. It provides useful preliminary findings, but is constrained by a lack of rigorous design and potential bias.

## Discussion

Our principle finding is that both stroke volume and peak velocity are significantly higher in iNPH patients compared to controls. However, there was notable heterogeneity in the results, as indicated by the I^2^ statistics for both stroke volume and peak velocity. While we have shown that PC-MRI confirms the hyperdynamic flow characteristic of iNPH, our narrative review has revealed that the utility of these parameters in predicting shunt responsiveness remains inconsistent. Results are limited by protocol variability and small study sizes. Flow studies play a valuable role in the diagnostic work-up of the disease, but rather than searching for threshold values to guide decision-making, it is more useful to assess a range of values that may reflect the varying likelihood of shunt success, considered alongside the whole clinical picture of each individual.

### Strengths and limitations

A significant strength of our study is the strict inclusion criteria for meta-analysis that allowed us to standardise data across studies. We focused only on the cerebral aqueduct as the region of interest (ROI), and focused only on stroke volume and peak velocity. This decision was based on the observations that net flow is prone to greater variability due to technical challenges in accurately measuring bidirectional flow.

We also acknowledge the limitations of this review, notably the heterogeneity of included studies in the narrative. Variability between studies is accounted for by different scanner protocols, outcomes measures, and differences in patient selection (Only 58% used Relkin as an international standard for diagnosis). While the classic Hakim’s triad of gait disturbance, cognitive impairment, and urinary incontinence is often cited, many studies include patients with only a subset of these symptoms. Some studies include *suspected* iNPH cases, justified by the notion that a definitive diagnosis can only be made based on clinical improvement after CSF removal. Although NOS quality assessment aims to identify such selection biases, the NOS itself remains a subjective measure.

Additionally, there is variability *within* studies. Many demonstrated relatively large standard deviations, which may be attributable to small sample sizes. However, it is worth considering the ethical and logistical challenges of designing a large-scale non-inferiority trial. The constraints of limited participants in these studies are often unavoidable. It is also worth noting that our study is the first meta-analysis of PC-MRI parameters in iNPH, making it difficult to directly compare our results with other pooled data.

### PC-MRI protocol variability

In cardiovascular imaging, standardised PC-MRI protocols for cardiovascular imaging are well established [4, 36]. No such standardisation exists for CSF analysis. 17 of the 18 studies included in our review reported the use of a standard protocol, (2D-PC Cine MRI. Supplementary Table 2) but even these had variations in encoding velocities, field of view, repetition time, echo time, and flip angle. Additionally, not all studies detailed their protocol, making it difficult to assess heterogeneity. Differences in MRI magnet strength and number of channels in the head coil can also introduce variability data interpretation [30]. The reviewed studies used a range of head coils (from 8-channel to 64-channel), each with a different signal-to-noise ratio, potentially introducing bias. Various velocity encoding (VENC) settings were also noted, which can also influence measurement accuracy.

CSF velocities are typically low, and the phase shifts in the signal are smaller at lower rates, making it difficult to distinguish true flow signal from background noise. Additionally, VENC is directly influenced by the amplitude, duration, and spacing of the gradient system, affecting the accuracy of velocity measurements. One study used both 1.5 T and 3 T machines, with higher field strengths providing better image resolution, but also more prone to artefacts [27]. Peak velocity measurements are less reliable at higher velocities due to the increased sensitivity, and artefacts are amplified by the limitations in temporal resolution. Stroke volume is generally less affected, because it represents the total volume of flow over a cardiac cycle, averaging out rapid fluctuations and artefacts. However, it can still be influenced by factors such as ROI selection.

Some protocols include an automatic or semi-automatic selection of the region, whereas others rely on a manual selection. When protocols rely on a hand-drawn selection, the derived values may be user-dependent. A larger ROI would underestimate flow parameters. We have attempted to assess this by including in our NOS assessment whether or not the selector is blinded to the study. Some authors have suggested that peak velocity may be a more accurate and repeatable parameter compared to stroke volume, since the value is ROI-independent [11, 24, 38]. By avoiding user-selected areas, variability in peak velocity may be more attributable to real pathological changes.

A 2019 editorial raised concerns about the accuracy and reproducibility of using PC-MRI to measure "bulk flow" the net movement of CSF through the aqueduct, citing the wide variability in estimates and ongoing debates about CSF dynamics [7]. Although these issues persist, we have addressed them by focusing solely on stroke volume and peak velocity, which are considered more reliable and less susceptible to artefacts than net flow measurements.

### Clinical implications

Our findings suggest that PC-MRI, particularly through the measurement of stroke volume and peak velocity, has clinical utility as part of the diagnostic work-up for iNPH. Elevated stroke volumes can help differentiate iNPH from a variety of other neurological diseases, and clinicians can use this as a non-invasive investigation to support an iNPH diagnosis.

However, the inconsistent predictive value of these parameters for determining shunt response limits their current clinical application in selecting patients for surgery. Clinicians may usefully refer to stroke volume and peak velocity for initial preoperative assessment, but this must be supplemented with other evaluations including gait analysis, neuropsychological testing, clinical symptom review, functional status and anaesthetic considerations, providing a full and comprehensive evaluation of shunt suitability. If future studies can refine and validate scanner protocols, PC-MRI could serve not only as a diagnostic aid but also as a means to monitor treatment efficacy and disease progression, potentially leading to more personalised and timely interventions for iNPH patients.

We suggest that it may be more effective to consider a range of values that reflect varying probabilities of shunt success. Bradley’s suggested stroke volume threshold of 42 µL could serve as a starting point, or a lower limit for shunt consideration. Upper limit values may represent a point beyond which the potential benefits of shunting are reduced, due to advanced disease progression and irreversible brain changes. In such cases the likelihood of achieving meaningful clinical improvement decreases. Wen-Jie He’s study showed that patients who did not improve after shunt placement had on average higher preoperative peak velocity values, a possible indication of an advanced disease stage [19]. Prompt intervention, when CSF flow metrics are only moderately elevated, may help achieve better results and avoid the complications associated with advanced iNPH where symptoms are more entrenched and irreversible.

### Next steps

One of the most significant challenges in PC-MRI research is the lack of standardised imaging protocols, which complicates efforts to establish consistent diagnostic thresholds. Future research should prioritise the development of guidelines for scanner settings, which may include for example flow parameter calculations, and region of interest (ROI) definitions. Establishing this is critical for improving the reliability and reproducibility of flow parameters across different clinical environments. Ideally, more robust data on the prognostic value of flow parameters would be provided by large-scale, multi-center prospective trials. However, recruiting large, well-defined cohorts of iNPH patients and healthy controls is challenging, and coordinating efforts across multiple centres requires significant logistical and financial resources.

The use of flow phantoms offers an effective alternative for validating PC MRI protocols. Significant differences in stroke volume measurements could highlight potential biases or errors in technique, set acceptable error limits, and improve the reliability of CSF flow measurements. A 2010 study by Wentland et al. utilised phantoms to investigate the accuracy and reproducibility of PC MRI. The study found that while the techniques were accurate for higher velocities, they significantly overestimated lower velocities [44]. Another technique validation study by Leutermer et al. found minimal intraobserver, interobserver, and intertrial variations. They demonstrated that their technique for measuring CSF flow was reliable, and confirmed accuracy across various flow rates, supporting its use for assessing CSF dynamics in clinical settings. The authors highlighted the importance of protocol standardisation [26].

Alongside the issues of validation, there is an increasing interest in how the glymphatic dysfunction is implicated in iNPH pathogenesis [32, 39]. CSF mediates the clearing of metabolic by-products from the brain, so impaired flow or decreased resorption may increase the risk of disease by metabolic or toxic stress. The importance of sleep, cardiac health, diet, and other unknown environmental factors are all likely to play a role in a functional glymphatic system. Algin et al. complimented CSF velocity measurements with venous velocity measured in the superior sagittal sinus, finding that venous velocity was significantly lower in iNPH than controls, and providing evidence for the theory of decreased CSF reabsorption in iNPH [1].

There is also a growing body of evidence that physiological CSF flow is not unidirectional [9, 11, 22, 34, 46, 47]. Net retrograde flow contributes to the accumulation of CSF within the ventricular system, parenchyma, and lymphatic vessels. Several of the studies reviewed here demonstrated retrograde flow in iNPH: Hamilton et al. found post-shunt patients had a decreased stroke volume at the pre-pontine cistern, with values trending towards the controls established in this study, suggesting a redistribution of CSF in iNPH patients [18]. Qvarlander et al. also found evidence for CSF redistribution, as stroke volume was lower than controls at the cervical level, and higher than controls at the aqueduct [31]. Chen et al. showed that backward flow had the highest diagnostic accuracy for identifying iNPH [11]. However, given the limited number of studies that incorporate retrograde flow into their analysis, further research is needed to assess and validate its diagnostic value. This would allow for a more comprehensive meta-analysis, and potentially enhance iNPH diagnosis.

Analysis using machine learning or neural networks to automate and optimise PC-MRI data have promising results [40, 42]. These approaches could identify subtle patterns in CSF flow that are not easily detected by current methods, potentially enhancing the diagnostic and prognostic capabilities of this modality. Integration of PC-MRI with other diagnostic tools such as clinical assessments, neuroimaging, and other biomarkers could further enhance its utility in clinical decision-making.

It is essential to contextualise the broader perspective of aqueductal CSF within a complex vascular system where blood flow serves as a key regulatory factor. The interaction between vascular pulsations and CSF movement significantly influences fluid distribution. Studies such as Qvarlander et al. have demonstrated how PC-MRI can simultaneously assess both vascular and CSF flows [31]. Approaches that integrate CSF and vascular flow metrics are necessary to further understand the interplay of intracranial fluid dynamics, and PC-MRI offers a modality that can assess flow in both components.

## Conclusions

This review adds to the growing evidence supporting PC-MRI as a useful tool for studying CSF flow dynamics in iNPH. The observation of increased stroke volume and increased peak velocity in iNPH patients across multiple studies is undeniable and consistent. However, the clinical utility of these findings for diagnosis, prognosis, or predicting surgical outcomes remains uncertain, due to the high variability between studies.

We emphasise the need for standardised PC-MRI protocols to fully explore its potential. Establishing a consensus on protocol specifics will enhance the reliability of PC-MRI findings, making it a more effective tool in diagnosing and managing iNPH. If these challenges can be addressed, PC-MRI could play a key role in the diagnosis and management of iNPH, potentially improving patient outcomes through better patient selection for surgical interventions.

Rather than seeking a definitive cut-off value, we suggest that interpreting stroke volume and peak flow values within a range may better reflect a patient’s condition and likelihood of shunt success. While PC-MRI shows promise as a non-invasive technique, future research demands involve larger sample sizes, longer follow-ups, and crucially, sensitivity and specificity analysis. This will clarify its diagnostic and prognostic value and support more informed clinical decision-making. Until then, We recommend that PC-MRI findings should be considered supplemental evidence rather than forming the basis of a clinical decision.

## Supplementary Information

Below is the link to the electronic supplementary material.

Supplementary file1 (PDF 138 KB)

Supplementary file2 (PDF 55.3 KB)

## Data Availability

No datasets were generated or analysed during the current study.

## Abbreviations

AD: Alzheimer's disease
CSF: Cerebrospinal fluid
IQR: Interquartile range
MRA: Magnetic resonance angiography
NF: Net flow
iNPH: Idiopathic normal pressure hydrocephalus
ICP: Intracranial pressure
NOS: Newcastle Ottawa Scale
PC-MRI: Phase contrast Magnetic Resonance imaging
PV: Peak velocity
ROI: Region of interest
SD: Standard deviation
SMD: Standardised mean difference
SV: Stroke volume
VENC: Velocity encoding

## Declarations

The study was supported by the Ministry of Health of the Czech Republic institutional grant no. NU23-04–00551.

## References

[1] Algin O, Hakyemez B, Parlak M (2010) The efficiency of PC-MRI in diagnosis of normal pressure hydrocephalus and prediction of shunt response. Acad Radiol 17(2):181–187. 10.1016/j.acra.2009.08.011 19910214 10.1016/j.acra.2009.08.011

[2] Alperin N (2004) MR-intracranial compliance and pressure: a method for noninvasive measurement of important neurophysiologic parameters. Methods Enzymol 386:323–349. 10.1016/S0076-6879(04)86016-6 15120260 10.1016/S0076-6879(04)86016-6

[3] Andersson J, Rosell M, Kockum K, Lilja-Lund O, Söderstr L (2019) Prevalence of idiopathic normal pressure hydrocephalus: A prospective, population-based study. PLoS One 14(5):e0217705. 10.1371/journal.pone.0217705 31141553 10.1371/journal.pone.0217705 PMC6541279

[4] ASCI Cct and CMR Guideline Working Group, Chan CW, Choi BW, ASCI, et al (2010) standardised practice protocol for cardiac magnetic resonance imaging: a report of the Asian society of cardiovascular imaging cardiac computed tomography and cardiac magnetic resonance imaging guideline working group. Int J Cardiovasc Imaging 2010:26. 10.1007/s10554-010-9708-y 10.1007/s10554-010-9708-y PMC2996543 20924794

[5] Balédent O, Henry-Feugeas MC, Idy-Peretti I (2001) Cerebrospinal fluid dynamics and relation with blood flow: a magnetic resonance study with semiautomated cerebrospinal fluid segmentation. Invest Radiol 36(7):368–377. 10.1097/00004424-200107000-00003 11496092 10.1097/00004424-200107000-00003

[6] Balédent O, Gondry-Jouet C, Meyer ME et al (2004) Relationship between cerebrospinal fluid and blood dynamics in healthy volunteers and patients with communicating hydrocephalus. Invest Radiol 39(1):45–55. 10.1097/01.rli.0000100892.87214.49 14701988 10.1097/01.rli.0000100892.87214.49

[7] Balédent O, Czosnyka Z, Czosnyka M (2019) “Bucket” cerebrospinal fluid bulk flow-is it a fact or a fiction? Acta Neurochir (Wien) 161(2):257–258. 10.1007/s00701-018-3731-5 30421028 10.1007/s00701-018-3731-5

[8] Bateman GA (2000) Vascular compliance in normal pressure hydrocephalus. AJNR Am J Neuroradiol 21(9):1574–1585 11039334 PMC8174849

[9] Borzage M, Ponrartana S, Tamrazi B, Gibbs W, Nelson MD, McComb JG, Blüml S (2018) A new MRI tag-based method to non-invasively visualize cerebrospinal fluid flow. Childs Nerv Syst 34(9):1677–1682. 10.1007/s00381-018-3845-3 29876643 10.1007/s00381-018-3845-3

[10] Bradley WG Jr, Scalzo D, Queralt J, Nitz WN, Atkinson DJ, Wong P (1996) Normal-pressure hydrocephalus: evaluation with cerebrospinal fluid flow measurements at MR imaging. Radiology 198(2):523–529 8596861 10.1148/radiology.198.2.8596861

[11] Chen CH, Cheng YC, Huang CY, Chen HC, Chen WH, Chai JW (2022) Accuracy of MRI derived cerebral aqueduct flow parameters in the diagnosis of idiopathic normal pressure hydrocephalus. J Clin Neurosci 105:9–15 36049363 10.1016/j.jocn.2022.08.018

[12] Daouk J, Chaarani B, Zmudka J et al (2014) Relationship between cerebrospinal fluid flow, ventricles morphology, and DTI properties in internal capsules: differences between Alzheimer’s disease and normal-pressure hydrocephalus. Acta Radiol 55(8):992–999. 10.1177/0284185113508112 24136984 10.1177/0284185113508112

[13] Enzmann DR, Pelc NJ (1991) Normal flow patterns of intracranial and spinal cerebrospinal fluid defined with phase-contrast cine MR imaging. Radiology 178:467–474 1987610 10.1148/radiology.178.2.1987610

[14] Feinberg DA, Mark AS (1987) Human brain motion and cerebrospinal fluid circulation demonstrated with MR velocity imaging. Radiology 163(3):793–799. 10.1148/radiology.163.3.3575734 3575734 10.1148/radiology.163.3.3575734

[15] Greitz D, Greitz T (1997) The pathogenesis and hemodynamics of hydrocephalus. A proposal for a new understanding. Int J Neuroradiol 3:367–375

[16] Greitz D, Franck A, Nordell B (1993Jul) On the pulsatile nature of intracranial and spinal CSF-circulation demonstrated by MR imaging. Acta Radiol 34(4):321–328 8318291

[17] Halperin J et al (2015) Practice guideline: Idiopathic normal pressure hydrocephalus: Response to shunting and predictors of response: Report of the Guideline Development, Dissemination, and Implementation Subcommittee of the American Academy of Neurology. Neurology 85(23):2063–2071 26644048 10.1212/WNL.0000000000002193 PMC4676757

[18] Hamilton RB, Scalzo F, Baldwin K et al (2019) Opposing CSF hydrodynamic trends found in the cerebral aqueduct and prepontine cistern following shunt treatment in patients with normal pressure hydrocephalus. Fluids Barriers CNS 16(1):2. 10.1186/s12987-019-0122-0 30665428 10.1186/s12987-019-0122-0 PMC6341759

[19] He WJ, Zhang XJ, Xu QZ et al (2022) Are preoperative phase-contrast CSF flow parameters ideal for predicting the outcome of shunt surgery in patients with idiopathic normal pressure hydrocephalus? Front Neurol 13:959450 Published 2022 Sep 27 10.3389/fneur.2022.959450 36237632 10.3389/fneur.2022.959450 PMC9552837

[20] Iseki C, Takahashi Y, Wada M, Kawanami T, Adachi M, Kato T (2014) Incidence of idiopathic normal pressure hydrocephalus (iNPH): a 10-year follow-up study of a rural community in Japan. J Neurol Sci 339:108–112 24656600 10.1016/j.jns.2014.01.033

[21] Jaraj D, Rabiei K, Marlow T et al (2014) Prevalence of idiopathic normal pressure hydrocephalus. Neurology 82(16):1449–1454. 10.1212/WNL.0000000000000342 24682964 10.1212/WNL.0000000000000342 PMC4001197

[22] Klarica M, Radoš M, Vukić M, Orešković D (2021) The physiology and pathophysiology of cerebrospinal fluid: new evidence. Croat Med J 62(4):307–309. 10.3325/cmj.2021.62.307 34472732 10.3325/cmj.2021.62.307 PMC8491049

[23] Kondziella D, Sonnewald U, Tullberg M, Wikkelso C (2008) Brain metabolism in adult chronic hydrocephalus. J Neurochem 106:1515–1524. 10.1111/j.1471-4159.2008.05422.x 18419769 10.1111/j.1471-4159.2008.05422.x

[24] Korbecki A, Zimny A, Podgórski P, Sąsiadek M, Bladowska J (2019) Imaging of cerebrospinal fluid flow: fundamentals, techniques, and clinical applications of phase-contrast magnetic resonance imaging. Pol J Radiol 84:e240–e250. 10.5114/pjr.2019.86881. Published 2019 May 13 31481996 10.5114/pjr.2019.86881 PMC6717940

[25] Lindstrøm EK, Ringstad G, Mardal K-A, Eide PK (2018) Cerebrospinal fluid volumetric net flow rate and direction in idiopathic normal pressure hydrocephalus, NeuroImage: Clinical, 20. ISSN 731–741:2213–1582. 10.1016/j.nicl.2018.09.006 10.1016/j.nicl.2018.09.006 PMC6154456 30238917

[26] Luetmer PH, Huston J, Friedman JA et al (2002) Measurement of cerebrospinal fluid flow at the cerebral aqueduct by use of phase-contrast magnetic resonance imaging: technique validation and utility in diagnosing idiopathic normal pressure hydrocephalus. Neurosurgery 50(3):534–544. 10.1097/00006123-200203000-00020 11841721 10.1097/00006123-200203000-00020

[27] Miskin N, Serulle Y, Wu W et al (2015) Post-Shunt Gait Improvement Correlates with Increased Cerebrospinal Fluid Peak Velocity in Normal Pressure Hydrocephalus: A Retrospective Observational Phase-Contrast Magnetic Resonance Imaging Study Int J Sci Study 3(8):48–54

[28] Moran PR, Moran RA, Karstaedt N (1985) Verification and evaluation of internal flow and motion. True magnetic resonance imaging by the phase gradient modulation method. Radiology 154(2):433–441. 10.1148/radiology.154.2.3966130 3966130 10.1148/radiology.154.2.3966130

[29] Naidich TP, Altman NR, Gonzalez-Arias SM, Imaging PCCMR (1993) Normal Cerebrospinal Fluid Oscillation and Applications to Hydrocephalus. Neurosurg Clin N Am 4(4):677–705. 10.1016/S1042-3680(18)30559-X 8241790

[30] Panman JL, To YY, van der Ende EL et al (2019) Bias Introduced by Multiple Head Coils in MRI Research: An 8 Channel and 32 Channel Coil Comparison. Front Neurosci 13:729. 10.3389/fnins.2019.00729 31379483 10.3389/fnins.2019.00729 PMC6648353

[31] Qvarlander S, Ambarki K, Wåhlin A et al (2017) Cerebrospinal fluid and blood flow patterns in idiopathic normal pressure hydrocephalus. Acta Neurol Scand 135(5):576–584. 10.1111/ane.12636 27388230 10.1111/ane.12636

[32] Reeves BC, Karimy JK, Kundishora AJ et al (2020) Glymphatic System Impairment in Alzheimer’s Disease and Idiopathic Normal Pressure Hydrocephalus. Trends Mol Med 26(3):285–295. 10.1016/j.molmed.2019.11.00847] 31959516 10.1016/j.molmed.2019.11.008 PMC7489754

[33] Relkin N, Marmarou A, Klinge P et al (2005) Diagnosing idiopathic normal-pressure hydrocephalus. Neurosurgery 57:S4-16. 10.1227/01.neu.0000168185.29659.c5 16160425 10.1227/01.neu.0000168185.29659.c5

[34] Ringstad G, Emblem KE, Eide PK (2016) Phase-contrast magnetic resonance imaging reveals net retrograde aqueductal flow in idiopathic normal pressure hydrocephalus. J Neurosurg 124(6):1850–1857. 10.3171/2015.6.JNS15496 26636385 10.3171/2015.6.JNS15496

[35] Shanks J, Markenroth Bloch K, Laurell K et al (2019) Aqueductal CSF Stroke Volume Is Increased in Patients with Idiopathic Normal Pressure Hydrocephalus and Decreases after Shunt Surgery. Am J Neuroradiol 40(3):453–459. 10.3174/ajnr.A5972 30792248 10.3174/ajnr.A5972 PMC7028668

[36] Stankovic Z, Allen BD, Garcia J, Jarvis KB, Markl M (2014) 4D flow imaging with MRI. Cardiovasc Diagn Ther 4(2):173–192. 10.3978/j.issn.2223-3652.2014.01.02 24834414 10.3978/j.issn.2223-3652.2014.01.02 PMC3996243

[37] Stecco A, Cassarà A, Zuccalà A, Anoaica, M., Genovese E, Car P, Panzarasa G, Guzzardi G, Carriero A. (2017). Quantitative analysis of cerebrospinal fluid dynamics at phase contrast cine-MRI: predictivity of neurosurgical "Shunt" responsiveness in patients with idiopathic normal pressure hydrocephalus. Journal of neurosurgical sciences. 64. 10.23736/S0390-5616.17.04092-9. 10.23736/S0390-5616.17.04092-9 28869371

[38] Tawfik AM, Elsorogy L, Abdelghaffar R, Naby AA, Elmenshawi I (2017) Phase-Contrast MRI CSF Flow Measurements for the Diagnosis of Normal-Pressure Hydrocephalus: Observer Agreement of Velocity Versus Volume Parameters. Am J Roentgenol 208(4):838–843 28140607 10.2214/AJR.16.16995

[39] Tan C, Wang X, Wang Y et al (2021) The Pathogenesis Based on the Glymphatic System, Diagnosis, and Treatment of Idiopathic Normal Pressure Hydrocephalus. Clin Interv Aging 16:139–153. 10.2147/CIA.S290709. Published 2021 Jan 15 33488070 10.2147/CIA.S290709 PMC7815082

[40] Tsou CH, Cheng YC, Huang CY et al (2021) Using deep learning convolutional neural networks to automatically perform cerebral aqueduct CSF flow analysis. J Clin Neurosci 90:60–67. 10.1016/j.jocn.2021.05.010 34275582 10.1016/j.jocn.2021.05.010

[41] Viechtbauer W (2010) Conducting meta-analyses in R with the metafor package. J Stat Softw 36(3):1–48. 10.18637/jss.v036.i03

[42] Vlasák A, Gerla V, Skalický P et al (2022) Boosting phase-contrast MRI performance in idiopathic normal pressure hydrocephalus diagnostics by means of machine learning approach. Neurosurg Focus 52(4):E6. 10.3171/2022.1.FOCUS21733 35364583 10.3171/2022.1.FOCUS21733

[43] Wells G A, Shea B, O’Connell D et al. (2013) The Newcastle-Ottawa Scale (NOS) for assessing the quality of nonrandomised studies in meta-analyses. Available at: http://www.ohri.ca/programs/clinical_epidemiology/oxford.asp, Accessed 14th May 2022

[44] Wentland AL, Wieben O, Korosec FR, Haughton VM (2010) Accuracy and reproducibility of phase-contrast MR imaging measurements for CSF flow. Am J Neuroradiol 31(7):1331–1336. 10.3174/ajnr.A2039 20203113 10.3174/ajnr.A2039 PMC3818526

[45] Witthiwej T, Sathira-ankul P, Chawalparit O et al (2012) MRI study of intracranial hydrodynamics and ventriculoperitoneal shunt responsiveness in patient with normal pressure hydrocephalus. J Med Assoc Thailand 95(12):1556–1562 23390787

[46] Yamada S (2021Aug 31) Cerebrospinal fluid dynamics. Croat Med J 62(4):399–410. 10.3325/cmj.2021.62.399 34472743 10.3325/cmj.2021.62.399 PMC8491047

[47] Yamada S, Ishikawa M, Ito H et al (2020) Cerebrospinal fluid dynamics in idiopathic normal pressure hydrocephalus on four-dimensional flow imaging. Eur Radiol 30(8):4454–4465. 10.1007/s00330-020-06825-6 32246220 10.1007/s00330-020-06825-6

[48] Yin LK, Zheng JJ, Zhao L et al (2017) Reversed aqueductal cerebrospinal fluid net flow in idiopathic normal pressure hydrocephalus. Acta Neurol Scand 136(5):434–439. 10.1111/ane.12750 28247411 10.1111/ane.12750

[49] Yousef MI, Abd El Mageed AE, Yassin AEN, Shaaban MH (2016) Use of cerebrospinal fluid flow rates measured by phase-contrast MR to differentiate normal pressure hydrocephalus from involutional brain changes. Egypt J of Radiol Nucl Med 47(3):999–1008

