# Supplementary material for: Volumes and velocities: Meta-analysis of PC-MRI studies in normal pressure hydrocephalus
Source: Acta Neurochir (Wien). 2024 Nov 19;166(1):463. doi: 10.1007/s00701-024-06333-2 (PMC11576626; doi:10.1007/s00701-024-06333-2)
Supplement: Supplementary file 1 — Supplementary file1 (PDF 138 KB) [file 701_2024_6333_MOESM1_ESM.pdf]

Supplementary table 1: Modified Newcastle Ottawa scale for cohort and case-control studies.

Quality criteria assessed for cohort studies:	
**Selection (total 4)**	
1) Representativeness of the exposed cohort	a) Very representative (four stars)<br>*Detailed description of inclusion and exclusion criteria,*<br>*Uses internationally recognised standardised guidelines (eg. Relkin, 2005)*
	b) Somewhat representative (three stars)<br>*Describes inclusion and exclusion criteria.*<br>*Uses standardised guidelines (eg. local guidelines)*
	c) Representativeness Poorly representative (two stars)<br>*Mentions inclusion and exclusion criteria, target population not clearly defined.*
	d) Selected group (one star)<br>*No inclusion or exclusion criteria*
	e) No description of the derivation of the cohort
**Comparability (total 2)**	
1) Comparability of cohorts on the basis of the design or analysis controlled for confounders	a) The study controls for two or more confounding factors<br>eg. age, gender, all scanned on same magnet strength (two stars)
	b) Study controls for one or more confounding factor (one star)
	c) Cohorts are not comparable on the basis of the design or analysis controlled for confounders
**Outcome (total 4)**	
1) Assessment of outcome	a) Independent blind assessment with record linkage (two stars)
	b) Record linkage only (one star)
	c) Self description or no description
2) Was follow-up long enough for outcomes to occur	a) Yes (one star)<br>*Follow up of three months or more*
	b) No / Unknown
3) Adequacy of follow-up of cohorts	a) All subjects accounted for (one stars)
	b) No statement, or subjects lost to follow up with no explanation
**Total possible score = 10**	

Quality criteria assessed for case-control studies:	
**Selection (total 4)**	
1) Adequacy of the definition of cases	a) Full definition of cases and independent validation (two stars)<br>*Detailed description of inclusion and exclusion criteria, and describing what the criteria were, uses internationally recognised standardised guidelines (eg. Relkin, 2005)*
	b) Adequate definition of cases (one star)<br>*Describes inclusion and exclusion criteria.*
	c) No definition of cases<br>*NPH diagnostic criteria not mentioned*
2) Adequacy of the definition of controls	a) Full definition of controls and population they were drawn from (two stars)
	b) Adequate definition of controls (one star)
	c) Inadequate definition / no definition of controls
**Comparability (2)**	
3) Comparability of cases and controls on the basis of design or analysis	a) Study controls for at least two variables (two stars)<br>*Eg. Age, sex, comorbidities, all scanned on same MRI machine*
	b) Study controls for at least one variable (one star)
	c) No description of controlling for variables
**Exposure and outcome (4)**	
4) Ascertainment of exposure	a) Secure record (two stars)<br>*Eg. Surgical record*
	b) Medical record only (one star)
	c) No description
5) Comparability of exposure	a) Method of ascertainment is same for both cases and controls (one star)
	b) Method of ascertainment is not the same or not described
6) Follow-up	a) Follow-up is the same for both groups, or difference between groups described and explained (one star)
	b) Follow-up between groups is not explained

Total possible score = 10
