# Supplementary material for: Volumes and velocities: Meta-analysis of PC-MRI studies in normal pressure hydrocephalus
Source: Acta Neurochir (Wien). 2024 Nov 19;166(1):463. doi: 10.1007/s00701-024-06333-2 (PMC11576626; doi:10.1007/s00701-024-06333-2)
Supplement: Supplementary file 2 — Supplementary file2 (PDF 55.3 KB) [file 701_2024_6333_MOESM2_ESM.pdf]

Author (Date)	MRI sequence	Magnet strength	Blinded researc	Relkin guideline	Selection (4)	Comparability (2	Outcome (4)	Total score (10)
Algin, O. (2010)	2D-PC cine	1.5T	Yes	No	3	2	4	9
Aslan, K. (2013)	2D-PC cine	3T	Yes	Yes	4	2	3	9
Chen, C. (2022)	2D-PC cine	3T	Yes	Yes	4	2	4	10
Daouk, J, (2014)	2D-PC cine	3T	Not stated	Yes	3	2	2	7
Gokul, U, R. (2018)	2D-PC cine	1.5T	Not stated	No	3	2	2	7
Hamilton, R.B. (2019)	2D-PC cine	3T	Yes	No	2	2	4	8
He, Wen-Jie (2020)	2D-PC cine	3T	Yes	Yes	4	2	3	9
He, Wen-Jie (2022)	2D-PC cine	3T	Yes	Yes	4	2	4	10
Lindstrom (2018)	2D-PC cine	3T	Yes	Yes	4	2	3	9
Miskin, N. (2015)	2D-PC cine	Both 1.5T and 3T	Not stated	No	3	1	3	7
Qvarlander, S (2017)	2D-PC cine	3T	Yes	Yes	4	2	4	10
Shanks, J (2019)	2D-PC cine	3T	Yes	Yes	4	2	4	10
Stecco, A. (2017)	2D-PC cine	Not stated	Not stated	No	3	1	2	6
Tawfik (2017)	2D-PC cine	1.5T	Yes	No	2	2	3	7
Yamada, S (2020)	4D flow (3D variant of 2D-cine)	3T	Not stated	No	3	2	2	7
Yin, L.K. (2017)	2D-PC cine	3T	Not stated	Yes	4	2	3	9
Yousef, M.I. (2016)	2D-PC cine	1.5T	Not stated	No	2	2	2	6
Witthiwej (2012)	2D-PC cine	3T	Not stated	No	2	1	2	5
